# *Echinococcus granulosus *sensu stricto hydatid cysts in human and livestock from northeastern Algeria: Molecular characterization based on *nad5* and *cox1* and fertility assessment

**DOI:** 10.1007/s00436-026-08703-4

**Published:** 2026-06-01

**Authors:** Amina Kheninef, Ilaria Bellini, Silvia Rondòn, Claudia Chiovoloni, Lynda Aissaoui, Serena Cavallero, Stefano D’Amelio

**Affiliations:** 1 Research Laboratory of Improvement and Development of Animal and Plant Production, Department of Biology and Animal Physiology, University Ferhat Abbas of Setif, Setif, Algeria; 2 Department of Public Health and Infectious Diseases, Sapienza University of Rome, Piazzale Aldo Moro 5, 00185 Rome, Italy

**Keywords:** *Echinococcus granulosus *sensu stricto, Mt-cox1, Mt-nad5, Cyst fertility, Algeria

## Abstract

Cystic Echinococcosis (CE) is a major helminthic zoonosis caused by the larval stage of *Echinococcus granulosus,* particularly highly endemic in several Mediterranean regions, including Algeria. Humans are accidental hosts, while animals such as livestock act as intermediate hosts. This study aimed to update the fragmented epidemiological picture of CE in Algeria, by combining the knowledge on cysts fertility, and protoscolex viability with molecular strains assignment. In particular, the genetic typing of *Echinococcus granulosus* from humans (*n* = 16), cattle (*n* = 15), sheep (*n* = 15), and goat (*n* = 1) collected in the northeastern Setif region, were performed using two partial mitochondrial markers used for a precise strains and variant call, the classic partial *cox1* and the recently developed *nad5*, used in this area for the first time. Results revealed a predominance of *E. granulosus *sensu stricto strain G1, with haplotype variants circulating in both humans and animals. Interestingly, G3 strain was reported only in a human patient, while G3 haplotype variants in both humans and animals. Fertility rate was lower but not negligible in cattle (18.2%), and high in sheep and humans (80.9% and 95.6%, respectively), and the viable cysts showed different anatomical distributions across species: in cattle, 43% were pulmonary and 57% hepatic; in sheep, 74.5% were located in the lungs and 25.5% in the liver. Such data highlights the crucial role of livestock in maintaining *E. granulosus* transmission in the area.

**Supplementary Information:**

The online version contains supplementary material available at 10.1007/s00436-026-08703-4.

## Introduction

Cystic Echinococcosis (CE) is a zoonotic parasitic disease caused by the larval stage of *Echinococcus granulosus,* common to humans and several animals. The parasite circulates mainly between canids (definitive hosts) and livestock (intermediate hosts), where it develops as larval hydatid cysts in organs like liver and lungs. Humans become accidental hosts through ingestion of vegetables or water contaminated with parasitic eggs eliminated by infected canids, or through contact with infected dogs, transporting eggs on their fur, mouth, or paws (from licking or rolling in contaminated soil) (Romig et al. 2015; Ma et al. 2021). Definitive hosts, in turn, become infected by ingesting cysts from the organs of infected intermediate hosts. This zoonotic cycle highlights the complex interactions between wildlife, domestic animals, and humans, and underscores the need for comprehensive surveillance and control measures to furtherly prevent the spread of this disease, listed by WHO among the Neglected Tropical Diseases, with prevalences varying significantly across countries and host species. In the Mediterranean region CE clearly remains a serious threat to public health and the economy across many countries, from coastal to internal regions. In European Mediterranean area, prevalence in livestock is generally low in some countries, such as France (0.00004% in sheep and 0.00003% in cattle) (Umhang et al. 2013). Conversely, significantly higher values are reported in countries like Italy, showing prevalence from 57.6% to 75% in sheep, from 41.5% to 67.1% in cattle in Sardinia and Sicily, respectively and 6% in dogs, and 0,2% in humans (Grosso et al 2012; Tamarozzi et al. 2020; Shamsi et al. 2022).

In the north-African Mediterranean region, Libya, Morocco and Tunisia are considered hyperendemic for CE, with high prevalence observed in rural areas. For instance, in Libya, more than 20% of stray and owned dogs (Buishi et al. 2005), up to 33.4% of sheep, between 1.0 and 13.9% of cattle and up to 18% of goats (Al-Khalidi 1998), 1.4–40.0% of dromedary camels (Ibrahem and Crellin 1998) are positive for CE. In Tunisia prevalence reach up to 40–100% in sheep over 2 years old (Lahmar et al. 1999, 2007), and a very high mean annual surgical incidence on humans of 12.7 per 100,000 inhabitants are reported (Chahed et al. 2010). Regarding the country specifically investigated in the present study, Algeria, CE remains widespread, with an average of prevalence of around 20% in stray dogs from urban and rural areas (Kohil et al. 2017), values ranging from 8.2% to 78.0% for sheep, and around 17% for cattle (Laatmna et al. 2019; Kheninef et al. 2024).

However, available data from the country are dispersed, geographically segregated, relying only on generally short sequences rather the full gene encoding *cox1, nad1* and *ef1alpha* markers, and reporting a large occurrence of *E. granulosus *sensu stricto, *E. canadensis* and *E. equinus* (Bardonnet et al. 2003; Zait et al. 2016; Laatmna et al. 2019; Kheninef et al. 2024).

This study aims to update the molecular epidemiological picture of CE in the northeastern area of Algeria (Setif province) using a combined approach, including the biological evaluation of cyst fertility and protoscolex vitality combined with the molecular characterization of strains. Genotyping of cysts were obtained using partial sequencing both the classic *cox1* and newly developed *nad5* markers*,* applied for the first time in this region. Cyst’s fertility and viability together with *E. granulosus s.s.* characterization of strains and variants support a better inference on how hosts contribute to parasite circulation and on the zoonotic risk (Hidalgo et al. 2019).

## Material and methods

### Sample collection and viability assessment

In 2023, 337 cysts were collected at controlled slaughterhouses in Setif province, Algeria: 187 from cattle (130 from lungs and 57 from livers), 126 from sheep (89 lungs and 37 livers), and one from a goat. Moreover, 23 human-derived cysts obtained post-surgical resection from patients admitted to the University Hospital Center of Setif (UHC), were analyzed, all from the liver. Immediately after sampling, the collected cysts were transferred to the research laboratory refrigerated. Fertility and viability were assessed following established protocols (Elissondo et al. 2008; Celik et al. 2021; Barazesh et al. 2018; Kheninef et al. 2024) and considering also anatomical location of the cyst.

### Molecular characterization of hydatid cysts and network analyses

Around 15% of cysts (*n* = 50) were selected for the molecular study, between protoscoleces for fertile cysts and germinal layers for sterile cysts, selecting cysts from both anatomical locations, when possible: 16 humans (all hepatic cysts), 16 bovine (12 hepatic and 4 pulmonary), 17 ovine (9 hepatic and 8 pulmonary) and 1 caprine (hepatic). After genomic DNA isolation (DNA Kit Isolate II, BIOLINE-meridian), two partial mitochondrial regions of *cox1* and the *nad5* were analysed according to the literature (Bart et al. 2006; Kinkar et al. 2018). In particular, primers for *cox1* 5′-TTTTTTGGCCATCCTGAGGTTTAT-3′) and reverse primer: (5′-TAACGACATAACATAATGAAAATG-3′), as previously described (Bart et al. 2006), while *nad5* was amplified with the forward primer (5′- GTTGTTGAAGTTGATTGTTTTGTTTG −3′) and the reverse primer (5′- GGA ACACCGGACAAACCAAGAA −3′), as previously described (Kinkar et al. 2018). Reliable sequences manually edited and trimmed with Trace implemented in MEGA 11 (Tamura et al. 2021) were compared to reference genotypes from the study area (Online Resources Tables 1 and 2 for comparative purposes). Alignments with ClustalW implemented in MEGA11 of the two mitochondrial regions were used to infer Median-Joining haplotypes Networks in PopART (Bandelt et al. 1999; Leigh & Bryant 2015), to evaluate the relationships of circulating genotypes, with respect to previously characterized samples collected in the region of study. All haplotypic variants identified were submitted to the NCBI database.

## Results

### Fertility and viability tests

Cysts fertility and viability were lower in cattle (18.2% and 61.7%, respectively), higher in sheep (80.9% and > 90%) and humans (95.6% and 100%). The single goat cyst was fertile and viable. Regarding the anatomical site of the vital cysts, for cattle the 43% were pulmonary cyst while the remaining 57% were hepatic, for sheep 74,5% was collected from the lungs and 25,5% from the liver; for humans and goat, all cysts were hepatic.

### Molecular genotyping and network analyses of haplotypes

**Fig. 1:**
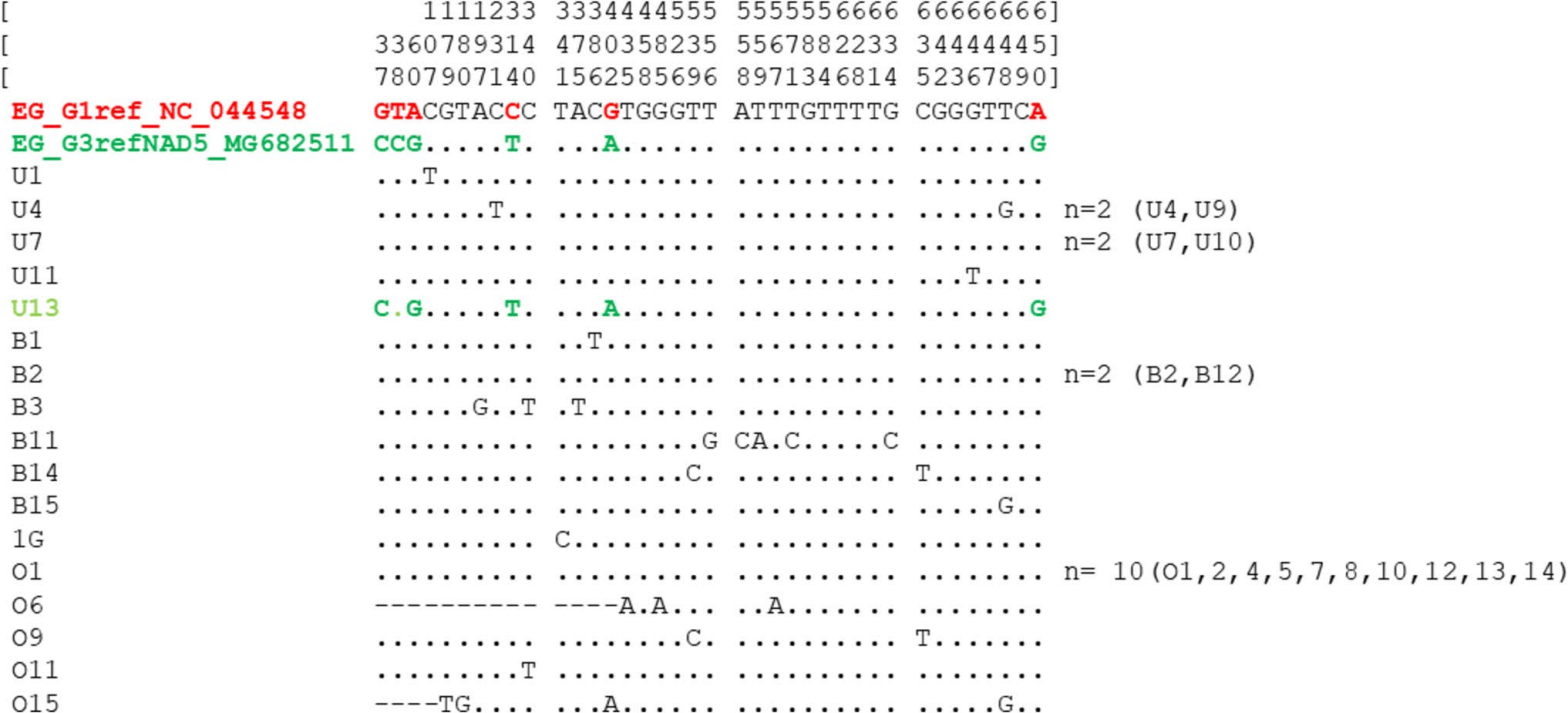
Variable sites at partial *nad5 E. granulosus s.s.* alignment of cysts here analysed together with two reference sequences for the main genotypes (G1 and G3). The number in columns indicated the alignment position of the variable site, with the number in rows (*n* =) indicate how many cysts showed the same polymorphism and corresponding specimens codes are indicated in parenthesis

**Fig. 2:**
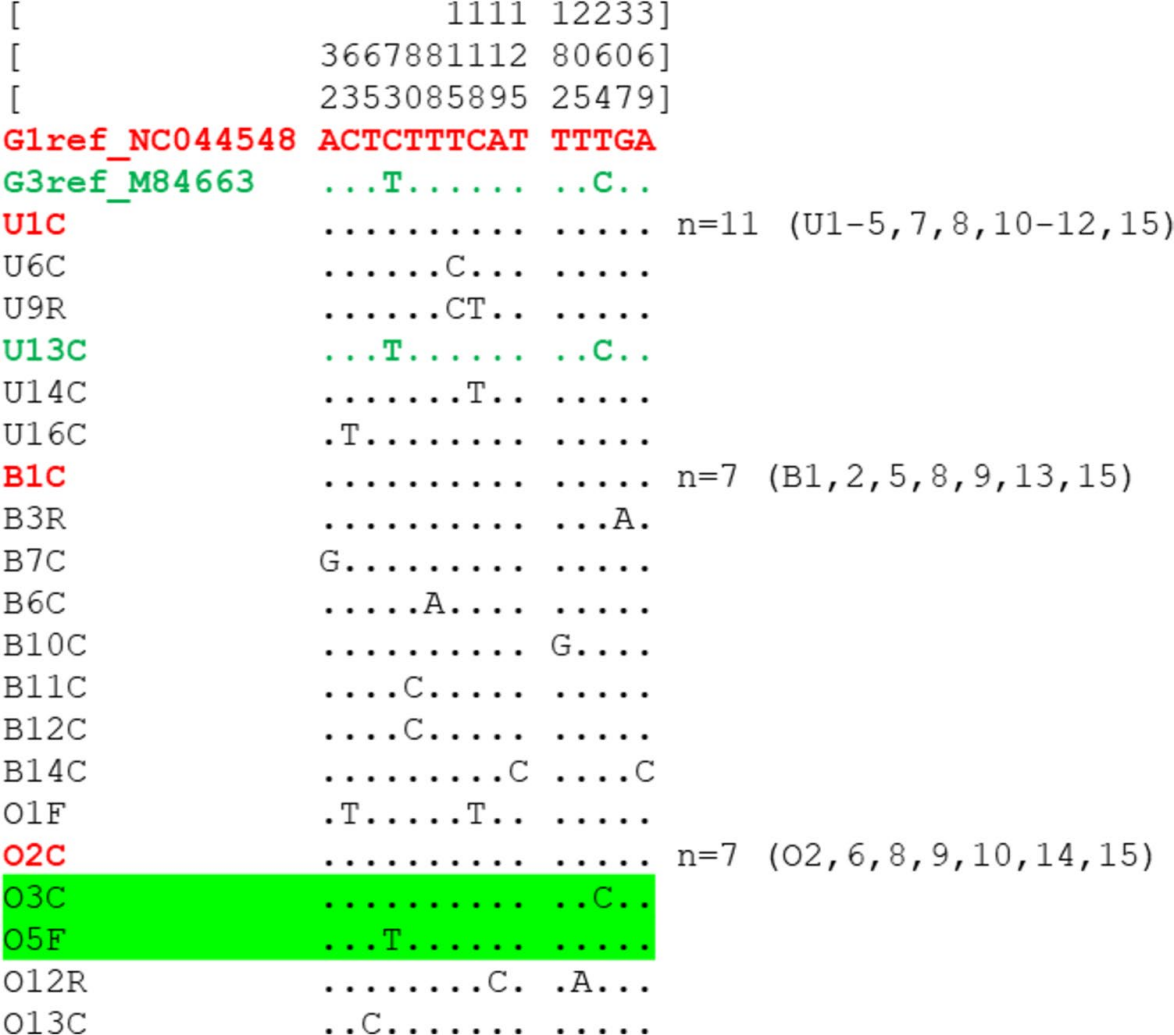
Variable sites at partial *cox1 E. granulosus* s.s. alignment of cysts here analysed together with two reference sequences for the main genotypes (G1 and G3). The number in columns indicated the alignment position of the variable site, with the number in rows (*n* =) indicate how many cysts showed the same polymorphism and corresponding specimens codes are indicated in parenthesis

Molecular genotyping was performed on a total of 50 samples, 47 of which provided good results at PCR amplification and sequences analyses. Data about the number of cysts collected, genotyped, fertility and viability regarding each host species and organs are presented in Table 1. The *nad*5 dataset consists of 28 good sequences, with 650 bp comprising 38 variable sites (Fig. 1), with Haplotype Diversity (HD) of 65%. Among them, 14 showed genotype G1 (2 from humans, 2 from bovine and 10 from ovine), one human sample was strictly related to G3 (showing 1 mismatch) and 14 were identified as haplotype variants (4 from humans, 5 from bovine, 4 from ovine, 1 from goat). Hosts showed similar number of haplotypes, with six occurring in humans and sheep samples and seven in bovine sample. GenBank accession numbers of the data here submitted for human derived samples are PX557021-27, for cattle PX557028-34, for the goat sample is PX557035 and for sheep are PX557036-47. For 15 cysts *nad5*-negative cysts (9 from humans, 1 ovine, 5 bovine), the strain assignment relies only on partial *cox*1 sequencing, implying uncertain genotyping. Regarding *cox1* sequencing results, 42 good sequences of 373 bp showing 15 variable sites were obtained, with Haplotype Diversity (HD) of 66%. Analyses of variable sites allowed to identify 25 specimens as belonging to *E. granulosus* s.s. genotype G1 (consisting of 11 human, 7 bovine, and 7 ovine), one specimen from a human case as belonging to genotype G3 and 16 variants (Fig. 2), with 4 originating from humans, 7 from bovine, and 5 from ovine, the latter including two variants with intermediate G1-G3 haplotypes. Hosts showed variable number of haplotypes, with six occurring in bovine, three in sheep, five in humans and 1 in goat sample. GenBank accession numbers for human derived samples are PX495113-28, for bovine are PX495129-42 and ovine PX495143-54.

**Table 1:** Details on *Echinococcus granulosus* sample analysed with a molecular approach in the present study. Host species from which cysts were collected, the specific specimen codes, the organ, the fertility and viability results (+ : fertile/vital; -:not fertle/not vital), the number of cyst characterized at molecular level, according to the two molecular markers used, *nad5* and *cox1*, and the strain assignment are reported. (mm: mismatches compared to G1 or G3)

Host species	code	Organ	Fertility and Viabiity	Molecular assay	Strain assignment
Human	U1-16	Liver	+ +	7/16 *nad5*	2G1, 2G1(1 mm), 2G1(2 mm), 1G3(1 mm)
				16/16 *cox1*	11G1, 3G1 (1 mm), 1G1 (2 mm),1G3
Sheep	O	Liver	+ +	7/17 *nad5*	6G1, 1G1(3 mm)
			+ +	5/17 *cox1*	3G1, 2G1(2 mm)
		Lungs	+ +	7/17 *nad5*	4G1, 1G1(1 mm), 1G1(2 mm), 1G1(4 mm)
			+ +	7/17 *cox1*	4G1, 3G1(1 mm)
Cattle	B	Liver	+ +	6/16 *nad5*	1G1, 1G1 (1 mm), 1G1(2 mm)
			- -		1G1 (1 mm), 1G1 (3 mm), 1G1 (5 mm)
			+ +	10/16 *cox1*	2G1, 1G1(1 mm)
			+ -		1G1
			- -		2G1, 4G1(1 mm)
		Lungs	+ +	1/16 *nad5*	1G1
			+ +	4/17 *cox1*	1G1(1 mm)
			- -		2G1, 1G1(1 mm)
Goat	1G	Liver	+ +	1/1 *nad5*	G1(1 mm)
				0/1 *cox1*

**Fig. 3:**
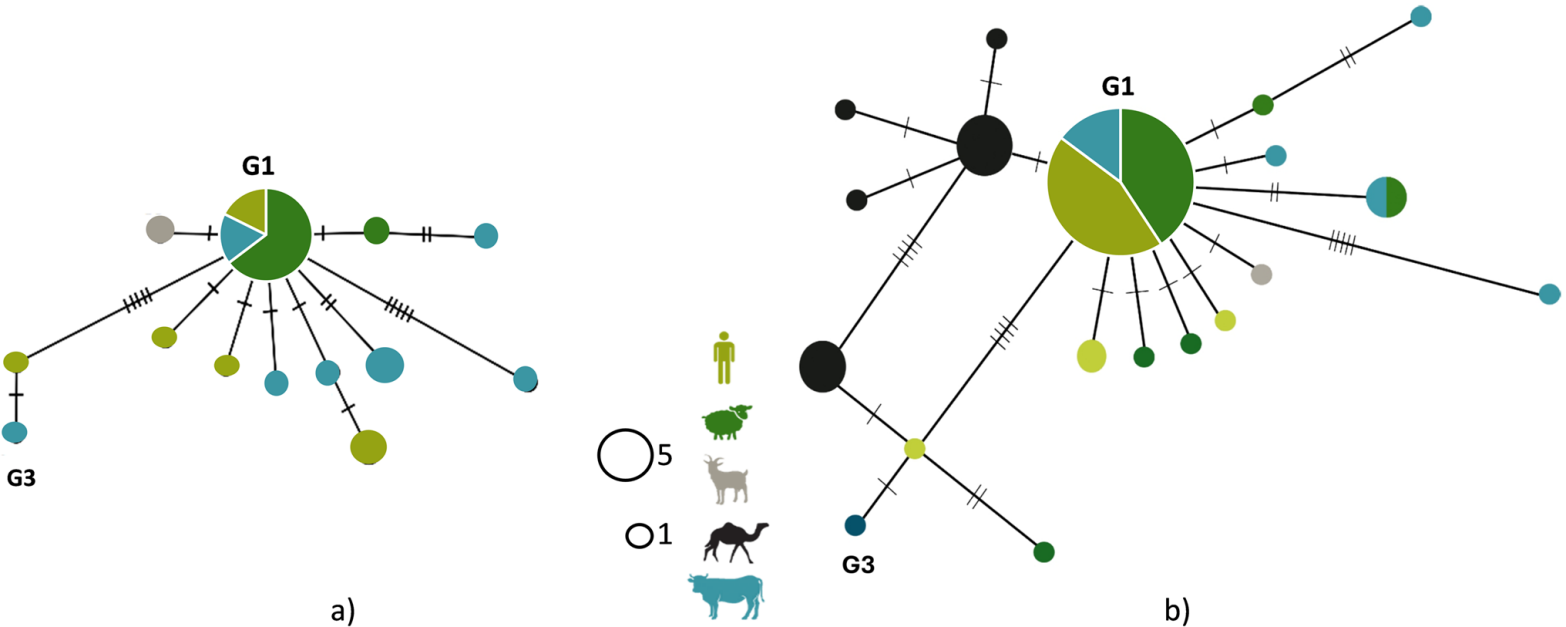
Median Joining networks of partial *nad5* of *E. granulosus* s.s. cysts here analysed (a) and in comparison to homologous sequences from Algeria retrieved from GenBank (b, Additional Table 1). The size of each circle is proportional to the number of specimens showing that specific haplotype, while transverse lines indicate SNPs. Colors of hosts symbols correspond to the colors indicated in the single circles and in pie chart

**Fig. 4:**
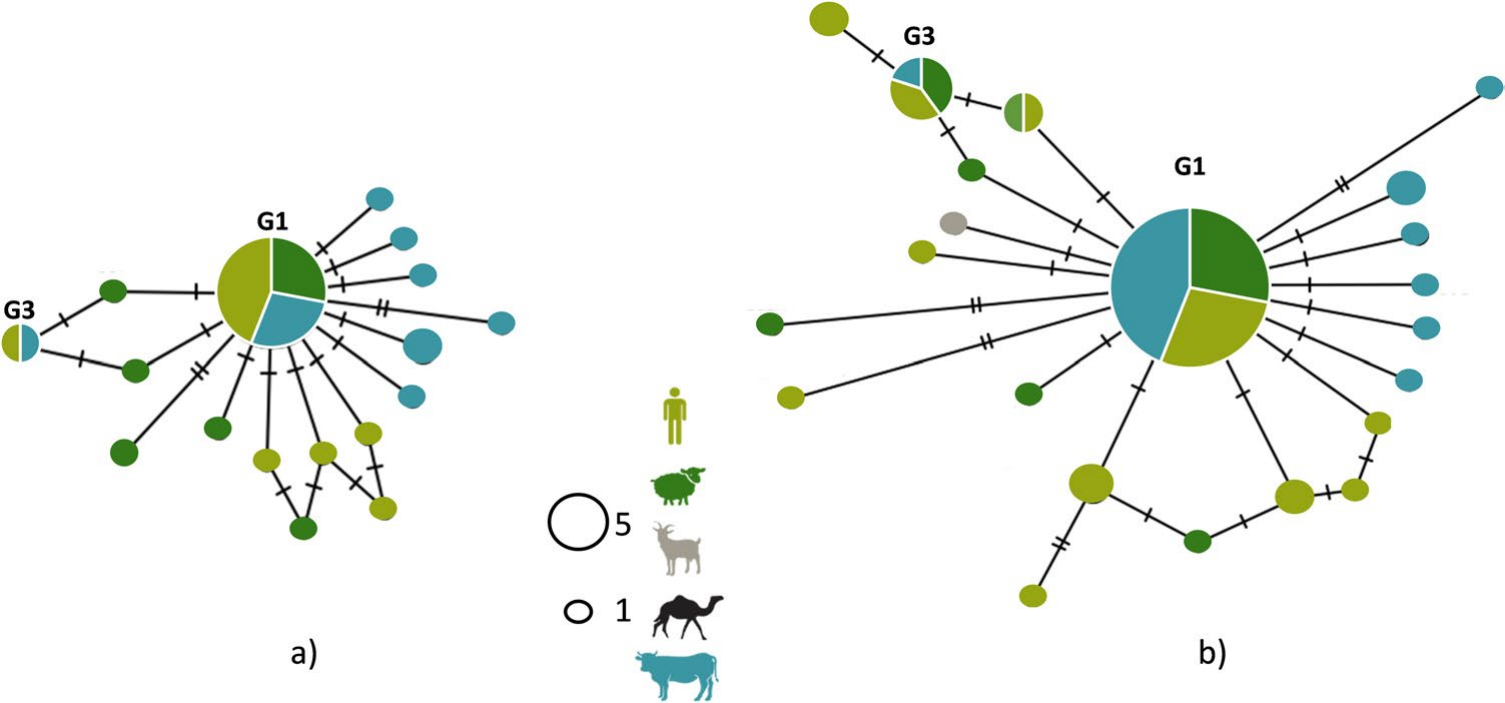
Median Joining networks of partial *cox1* of *E. granulosus* s.s. cysts here analysed (a) and in comparison to homologous sequences from Algeria retrieved from GenBank (b, Additional Table 1). The size of each circle is proportional to the number of specimens showing that specific haplotype, while transverse lines indicate SNPs. Colors of hosts symbols correspond to the colors indicated in the single circles and in pie chart

MJ networks aimed to explore the genealogy and evolutionary relationships of haplotypes in both markers from the data here analysed, as well as in comparison to GenBank sequences from intermediate hosts from the same geographical area (Algeria), clearly indicated G1 in the central star-like position for both markers (Fig. 3 and 4).

In *nad5* networks, most of the ovine sequences cluster in the main G1 group (Fig. 3a) and all remaining variants circulating in bovine, goat and in humans are independently related to G1, except for one human case which is closely related to G3. In Fig. 3b, strains found in camels from southern Algeria related to the pathway linking G1 and G3 strains, including in bridging squared part of the picture also two human derived sequences, both closely related to G3.

A similar pattern is observed for *cox1* networks (Fig. 4a and 4b), and the ovine and human haplotype variants are more closely related to each other than to bovine variants.

## Discussion

The molecular characterization and genetic diversity of *Echinococcus granulosus* sensu lato have not been widely studied in Algeria, despite the importance of evidence in revealing crucial knowledge into the distribution of genotypes and epidemiology of this parasitic zoonosis.

In the present study, the presence of *E. granulosus* cysts were evaluated in livestock and human intermediate hosts, to explore their genetic variability and accounting for their putative active role in parasite transmission, according to the evidence on fertility and viability of hydatid cysts circulating in north-eastern region of Algeria.

Here, two mitochondrial molecular markers were used in combination, *nad5* and *cox1*, as *nad5* enabled to make a clear distinction between G1 and G3 and *cox1* was massively used notably in Algeria enabling comparison with previous data, in order to update the epidemiological picture of CE in intermediate hosts. The parasite circulation is confirmed in the area, never investigated before with such approach, and a large domination of G1 was described. A moderate-to high HD was obtained for both markers, in agreement with recent data from Algeria and other endemic countries (Samari et al. 2022), suggesting a sustained presence of CE in the area. The majority of G1 and G1-derived variants were observed in animal populations: their occurrence in all animal species investigated and in humans, supported prior findings and underlining the parasite’s adaptability to a range of hosts being more generalist. The circulation of G1 in numerous host species is consistent with prior evidence: Laatmna et al. (2019) classified G1 as the main genotype in cattle from the steppe area of Djelfa, central northern Algeria. Samari et al. (2022) discovered G1 in dromedary camels from the extreme Sahara areas of Tindouf and Illizi. These data illustrate the extensive prevalence of the G1 genotype throughout varied ecological areas in Algeria, spanning from the north vegetated (not-desertic) area of the country to the southern Sahara.

The G3 genotype was detected exclusively in one human derived sample in the current research, in agreement with recent findings from El Oued region of Algeria, at the beginning of the desertic part of the country (Kinkar et al. 2018). This narrow host specificity for the G3 genotype in humans, as contrast to its minimal occurrence in animals, implying a potential niche adaptation or epidemiological importance particular to human hosts. Moreover, its close relationship with camel-derived sequences is of particular interest.

Together with biological implications related to *E. granulosus s.s.* strain identity, the evaluation of fertility and viability of the hydatid cysts in the intermediate hosts contribute to a better understanding of the real role on the parasite transmission of such hosts. In fact, livestock represent the main source of transmission to the final host in countries devoted to pastoralism, thanks to the prey-predator relationship and the ingestion of fertile cysts by the predators. In fact, the fertility of the hydatid cyst may vary according to geography, animal species, anatomical cysts locations, size, and genotype of the cyst, likely due to the immunological host response (Khan et al. 2001; Hajimohammadi et al. 2022).

In Iran, a high fertility rate was observed in the liver cysts isolated from sheep, followed by lung cysts in goats and camels, while unfertile cysts were reported in cattle (Hajimohammadi et al. 2022).

Data from humans here analysed showed very high cysts fertility and viability, similarly to data from Iraq, mainly involving G1 or G3 haplotypes (Issa et al. 2022), even if recently a fertility rate of around 50% was recorded with only G1 mentioned, according to 12S sequencing (Benmarce et al. 2026). Despite the potential presence of fertile and viable *Echinococcus* cysts in humans, the species does not contribute to the transmission dynamics of the parasite. Humans act as accidental, dead-end hosts and thus have no epidemiological relevance in the maintenance of the life cycle.

Here, the lowest but not negligible values of fertility and viability were observed in cattle, and higher values were observed in sheep derived cysts.

Considering the epidemiological data of CE available from Algeria and other aspects such as the estimated number of sheep in the country of around 20 million (FAO 2014) and a presumptive regular consumption of offal by the dogs, the accurate strains and variants identification of hydatid cysts together with evaluation of cysts fertility are of major epidemiological importance, contributing to understand strains circulation, intermediate role in parasite transmission and potentially tracing haplotypic differences in infectivity to humans.

## Supplementary Information

Below is the link to the electronic supplementary material.

Supplementary file1 (DOCX 21 KB)

## Data Availability

Data is provided along the manuscript or supplementary information files.
