# Supplementary material for: Echinococcus granulosus sensu stricto hydatid cysts in human and livestock from northeastern Algeria: Molecular characterization based on nad5 and cox1 and fertility assessment
Source: Parasitol Res. 2026 Jun 1;125(1):93. doi: 10.1007/s00436-026-08703-4 (PMC13447339; doi:10.1007/s00436-026-08703-4)
Supplement: Supplementary file 1 [file 436_2026_8703_MOESM1_ESM.docx]

**SUPPLEMENTARY MATERIAL**

**Parasitology Research**

***Echinococcus granulosus sensu stricto* hydatid cysts in human and livestock from northeastern Algeria: molecular characterization based on *cox1* and *nad5* and fertility assessment.**

Amina Kheninef^1^, Ilaria Bellini^2*^, Silvia Rondòn^2^, Claudia Chiovoloni^2^, Lynda Aissaoui^1^, Serena Cavallero^2^, and Stefano D’Amelio^2^

1. Research Laboratory of Improvement and Development of Animal and Plant Production, Department of Biology and Animal Physiology, University Ferhat Abbas of Setif, Setif, Algeria

2. Department of Public health and infectious diseases of Sapienza University of Rome, Piazzale Aldo Moro 5, 00185 Rome Italy

*corresponding author: Ilaria Bellini ORCID: 0009-0002-2218-7550 [ilaria.bellini@uniroma1.it](mailto:ilaria.bellini@uniroma1.it)

**Supplementary Table 1**: List of sequences material used for comparative purposes on *Echinococcus granulosus sensu stricto,* reporting the molecular region, the genotype, the host species, the list of accession numbers and the authors.

| **Region** | **Genotype** | **Host**  **species** | **GenBank**  **Accession**  **Number** | **Authors** |
| --- | --- | --- | --- | --- |
| COX1 | G1 | Sheep | MG808287  MG808290  MG808293  MG808294  MG808296  MG808298  MG808308  MG808313  MG808315  MG808316  MG808317  MG808327  MG808328  MG808334  MG808337  MG808338  MG808339  MG808340 | Laatamna et al. 2019 |
| COX1 | G1 | Cattle | MG808292  MG808307  MG808318  MG808324  MG808330  MG808343 | Laatamna et al. 2019 |
| COX1 | G3 | Sheep | MG808314  MG808332 | Laatamna et al. 2019 |
| Partial genome  (Including  nad5 & cox1) | G1 | *Homo sapiens* | MG672284  MG672285  MG672286  MG672287  MG672288  MG672128  MG672289  MG672290  MG672291  MG672292  MG672293 | Kinkar et al. 2018a |
| Partial genome  (Including  nad5 & cox1) | G3 | *Homo sapiens* | MG682544 | Kinkar et al. 2018b |
| COX1 | G1 | *Homo sapiens* | KR349027  KR349028  KR349029  KR349030  KR349031  KR349032  KT316341  KR381826 | Zait et al. 2016 |
| COX1 | G1 | Bovine | KR349033 | Zait et al. 2016 |
| COX1 | G1 | Goat | KR349034 | Zait et al. 2016 |
| COX1 | G3 | *Homo sapiens* | KR349035  KR349036 | Zait et al. 2016 |

**Supplementary Table 2**: List of sequences material used for comparative purposes on *Echinococcus granulosus sensu stricto,* reporting the molecular region, the genotype, the host species, the list of accession numbers and the authors.

| **Sequence** | **Genotype** | **Host**  **species** | **GenBank**  **Accession**  **Number** | **Authors** |
| --- | --- | --- | --- | --- |
| NAD5 | G1 | *Camelus dromedarius* | ON049457  ON049458  ON049459  ON049460  ON049461  ON049462  ON049463  ON049464  ON049465  ON049466 | Samari et al. 2022 |
| NAD5 | G3 | *Camelus dromedarius* | ON049438  ON049439  ON049440  ON049441  ON049442 | Samari et al. 2022 |
| Partial genome  (Including  NAD5 & COX1) | G1 | *Homo sapiens* | MG672284  MG672285  MG672286  MG672287  MG672288  MG672128  MG672289  MG672290  MG672291  MG672292  MG672293 | Kinkar et al. 2018a |
| Partial genome  (Including  nad5 & cox1) | G3 | *Homo sapiens* | MG682544 | Kinkar et al. 2018b |
